# COVID-19 pandemic and involuntary admitted patients in a Psychiatric Service

**DOI:** 10.1192/j.eurpsy.2023.1649

**Published:** 2023-07-19

**Authors:** C. Portela, C. Oliveira, M. Gonçalves

**Affiliations:** Hospital de Magalhães Lemos, Porto, Portugal

## Abstract

**Introduction:**

The COVID-19 pandemic had a major impact on mental health globally, resulting in a need for adaptation of mental health services. The psychosocial consequences of this crisis, such as psychological stress, reduction of community care and social support, are known factors that increase the risk of psychiatric decompensation. Compulsory admission is the last line of intervention in individuals who suffer from severe mental disorders and refuse treatment, based on the principles of therapeutic need and social protection. In Portugal, the last law regulating the compulsory admission is in forme since 2004 (Law 36/98 of 24 July), and configures this measure as a hospitalization by court order, happening the same in other European countries. The literature shows that in 2020 there was a significant increase in the proportion of involuntary inpatient admissions for all psychiatric diagnosis.

**Objectives:**

This study aims to assess the impact of the COVID-19 pandemic on involuntary admissions to an acute psychiatric service.

**Methods:**

Socio-demographic and clinical data were collected from electronic medical records. A retrospective observational study of patients who were admitted in a General Psychiatric Unit of Hospital Magalhães Lemos between March 2019 and February 2021 was conducted. The characteristics of patients admitted before the pandemic (March 2019 to February 2020) and after (March 2020 to February 2021) were compared statistically.

**Results:**

A total of 850 patient admissions were obtained, of which 28% were involuntary. The authors expect to find differences between involuntary inpatient admissions before and after the COVID-19 pandemic in proportion of patients, socio-demographic and clinical factors.

**Conclusions:**

The findings of this study will likely show an increase in involuntary admissions during the pandemic, in agreement with current knowledge. More studies are needed to assess the long-term impact of the pandemic on mental health.

**Disclosure of Interest:**

None Declared

